# Cavity Field-Driven Symmetry Breaking and Modulation of Vibrational Properties: Insights from the Analytical QED-HF Hessian

**DOI:** 10.1021/acs.jctc.5c00680

**Published:** 2025-10-03

**Authors:** Alberto Barlini, Andrea Bianchi, Jan Haakon M. Trabski, Julien Bloino, Henrik Koch

**Affiliations:** † 19004 Scuola Normale Superiore, Piazza dei Cavalieri 7, Pisa 56126, Italy; ‡ Department of Chemistry, 8018 Norwegian University of Science and Technology, NTNU, Trondheim 7491, Norway

## Abstract

In this work, we present the analytical derivation and implementation of the quantum electrodynamics Hartree–Fock Hessian. We investigate how electronic strong coupling influences molecular vibrational properties, applying this framework to formaldehyde, *p*-nitroaniline, and adamantane. Our analysis reveals cavity-induced changes in vibrational frequencies and intensities. Additionally, we show how the quantum electromagnetic field breaks molecular symmetry, activating previously forbidden infrared transitions. Our findings highlight the potential of coupling photonic and electronic degrees of freedom to control and modulate molecular vibrational properties in the ground state.

## Graphical abstract

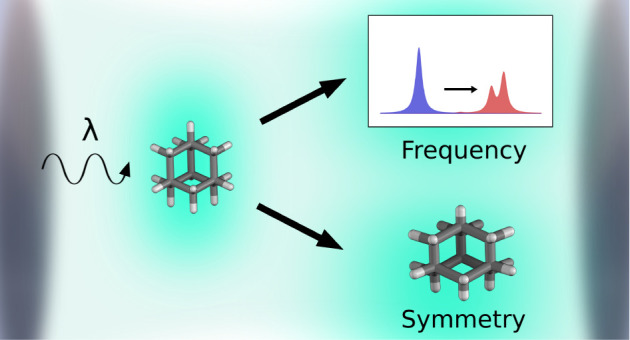

## 1 Introduction

Computing the analytical Hessian is essential for an accurate and numerically stable description of the vibrational properties and for identifying stationary points on the potential energy surface (PES), enabling the detailed analysis of vibrational spectra and providing deeper insight into chemical reaction pathways. Significant effort has been directed toward developing theoretical methods and algorithms to evaluate the Hessian using various approximate wave function methods, which rely on the analytical second derivatives of the electronic energy.^1–11^

In the strong light-matter coupling regime, interactions between molecules and confined electromagnetic fields form hybrid light-matter states known as polaritons. Depending on whether the cavity mode is resonant with an electronic or a vibrational transition, the system is described as being in the electronic (ESC) or vibrational strong coupling (VSC) regime, respectively. To describe these systems, two different approaches have been developed: the cavity Born–Oppenheimer approximation (CBOA)^12–15^ and the so-called polaritonic approaches.^16–18^ The CBOA is particularly well suited for investigating phenomena under VSC. In this framework, the cavity potential energy surface (cPES) is constructed by treating nuclear and photonic coordinates on an equal footing for a fixed electronic state. As a result, the formation of vibro-polaritons arises naturally from the curvature of the cPES. In contrast, in all polaritonic approaches, photonic and electronic degrees of freedom are coupled, while nuclear degrees of freedom are treated separately. This formulation is more suitable to investigate the ESC regime, as it allows for the explicit treatment of mixed electronic-photonic excited states. However, this framework precludes the description of resonant vibro-polaritonic states, which require a unified treatment of nuclear and photonic degrees of freedom. Recent studies have explored cavity-induced effects on molecular geometries and infrared (IR) spectra under VSC^19–21^ and ESC^22,23^ regimes. However, the theoretical framework and analytical evaluation of the vibrational properties in the ESC regime remain unexplored.

Here, we present the derivation and implementation of the analytical quantum electrodynamics Hartree–Fock (QED-HF)^16^ Hessian and apply it to cavity-molecule systems to investigate how light-matter interactions influence vibrational properties and IR spectra under ESC. The QED-HF method does not explicitly account for the cavity frequency. All physical observables—such as the energy, dipole moment, vibrational frequencies, and other molecular properties—depend exclusively on the light-matter coupling strength. Our approach enables us to assess how cavity-induced changes in the electronic density reshape the potential energy surface, thereby altering the normal modes and systematically shifting the vibrational frequencies. Moreover, we focus only on single-molecule effects, neglecting any collective phenomena mediated by the cavity.

We chose three prototypical systems to explore different aspects of the cavity effects, starting from a planar system, formaldehyde, a standard benchmark molecule. *p*-Nitroaniline (PNA) is a typical ″push–pull″ molecule with an electron-donor group NH_2_ and an acceptor group NO_2_. Given the high sensitivity of this system due to its large dipole moment, we expect significant cavity-induced modifications in its vibrational properties. Finally, adamantane, with its rigid and spherical-top structure and *T*_d_ symmetry, provides an ideal system for exploring symmetry-breaking effects induced by the cavity field. As the quantum electromagnetic field modifies the electronic potential energy surface, nuclear motion is consequently altered, leading to changes in the IR spectra. Our results reveal how cavity interactions modify vibrational modes, leading to frequency shifts and intensity modulation in the IR spectra. This paper is organized as follows: in Section 2, we derive the QED-HF Hessian expression. In Section 3, we discuss its implementation and validation. In Section 4, we report the results for the investigated systems. Finally, in Section 5, we provide our concluding remarks.

## 2 Theory

### 2.1 QED Hamiltonian

In the Born–Oppenheimer approximation, the interaction between light and matter in the dipole approximation is described by the Pauli-Fierz Hamiltonian, which, in its length-gauge form, is given by^24,25^

1 HPF=HM+∑αωαbα†bα+∑α∑pqωα2(λα·d)pq(bα+bα†)Epq+12∑α∑pqr(λα·d)pr(λα·d)rqEpq+12∑α∑pqrs(λα·d)pq(λα·d)rsepqrs

where *H*_M_ denotes the molecular Hamiltonian

2 $$H_{M}=T^{\mathrm{nuc}}+V^{\mathrm{nuc}}+H^{\mathrm{el}}$$

*T* ^nuc^ is the nuclear kinetic energy, *V* ^nuc^ is the nuclear repulsion energy, and *H* ^el^ is the electronic Hamiltonian

3 $$H^{\mathrm{el}}=\underset{pq}{\sum }h_{pq}E_{pq}+\frac{1}{2}\underset{pqrs}{\sum }g_{pqrs}e_{pqrs}$$

with *h*_*pq*_ and *g*_*pqrs*_ denoting the one- and two-electron integrals, respectively. In eqs 1 and 3, the electronic operators are defined as follows

4 $$\begin{array}{l} E_{pq}=\underset{\sigma }{\sum }a_{p\sigma }^{†}a_{q\sigma },\\ e_{pqrs}=E_{pq}E_{rs}-\delta_{rq}E_{ps} \end{array}$$

where the indices *p*, *q*, *r*, and *s* refer to molecular orbitals, and $a_{p\sigma }^{†}$ and *a*_*pσ*_ are the creation and annihilation operators for an electron in orbital *p* with spin σ. The strong-coupling Hamiltonian in eq 1 includes the quantum electromagnetic field energy, the light-matter interaction explicitly correlating the field and electrons, and the dipole self-energy terms, which ensure the Hamiltonian is bounded from below.^26^ Here, the annihilation and creation operators *b*_α_ and $b_{\alpha }^{†}$ are introduced for photons in mode α, with frequency ω_α_ and polarization vector **λ**_α_

5 $$\lambda_{\alpha }=\sqrt{\frac{2\pi }{\varepsilon_{r}V_{\alpha }}}\varepsilon_{\alpha }$$

where *ε*_*r*_, *V*_α_, and ***ε***_α_ are the relative permittivity, the quantization volume, and the polarization unit vector, respectively. The total dipole moment operator in eq 1 is defined as

6 $$\underset{pq}{\sum }d_{pq}=\underset{pq}{\sum }\left(d_{pq}^{\mathrm{el}}+\frac{d_{pq}^{\mathrm{nuc}}}{N_{e}}\delta_{pq}\right)E_{pq}$$

where $d_{pq}^{\mathrm{el}}$ and $d_{pq}^{\mathrm{nuc}}$ represent the electronic and nuclear dipole moments, respectively, and *N*_*e*_ is the number of electrons.

### 2.2 QED-HF Model

In the QED-HF model, the wave function is expressed as

7 |QED−HF⟩=|HF⟩⊗|P⟩

where |HF⟩ denotes a single Slater determinant, and |P⟩ is the photon state defined as

8 $$\left|P\right\rangle =\underset{n}{\sum }\underset{\alpha }{\prod }{\left(b_{\alpha }^{†}\right)}^{n_{\alpha }}\left|0\right\rangle c_{n}$$

where |0⟩ represents the photonic vacuum state, *c*_**n**_ are the coefficients for the expansion in photon number states, and **n** = (*n*_1_, *n*_2_,···) corresponds to the state with *n*_α_ photons in mode α. The orbitals in the HF reference are optimized using an orthogonal transformation, defined as exp­(-κ), where κ is an antisymmetric one-electron operator, and the QED-HF energy is minimized with respect to the photon coefficients by diagonalizing the photonic Hamiltonian through an orthogonal coherent state transformation as described by Haugland et al. in ref 16

9 $$U=\underset{\alpha }{\prod }\exp\left(-\frac{\lambda_{\alpha }\cdot \left\langle d\right\rangle }{\sqrt{2\omega_{\alpha }}}\left(b_{\alpha }-b_{\alpha }^{†}\right)\right)$$

where

10 ⟨d⟩=⟨HF|d|HF⟩

is the expectation value of the total dipole moment operator **d**. The transformation of the Hamiltonian in eq 1 gives

11 H=U†HPFU=Hel+∑αωαbα†bα+∑α∑pqωα2(λα·(d−⟨d⟩))pq(bα+bα†)Epq+12∑α∑pqr(λα·d)pr(λα·d)rqEpq−∑α∑pq(λα·⟨d⟩)(λα·d)pqEpq+12∑α∑pqrs(λα·d)pq(λα·d)rsepqrs+12∑α(λα·⟨d⟩)2

In the coherent-state basis, the reference wave function becomes

12 $$\left|R\right\rangle =\underset{\alpha }{\prod }\exp\left(-\frac{\lambda_{\alpha }\cdot \left\langle d\right\rangle }{\sqrt{2\omega_{\alpha }}}\left(b_{\alpha }-b_{\alpha }^{†}\right)\right)\exp\left(-\kappa \right)\left|\mathrm{QED}\text{−}\mathrm{HF}\right\rangle$$

and the QED-HF energy is invariant with respect to the choice of molecular origin, even for charged molecules. The energy expression may be written as

13 $$E_{\mathrm{QED‐HF}}=E_{\mathrm{HF}}+\underset{\alpha }{\sum }\underset{ai}{\sum }{\left(\lambda_{\alpha }\cdot d_{ai}\right)}^{2}$$

where the labels *i* and *a* indicate occupied and virtual orbitals, respectively.

### 2.3 Geometrical Derivatives of the QED Hamiltonian

Consider a molecular system in the neighborhood of some reference geometry **R**_0_ described by the reference wave function given in eq 12. The corresponding molecular orbitals are expanded in a set of atomic orbitals centered on the nuclei,

$$\phi_{p}\left(R_{0}\right)=\underset{\mu }{\sum }C_{p\mu }\;\chi_{\mu }\left(R_{0}\right)$$ 14

This definition can be extended to other molecular geometries by introducing the symmetrically orthonormalized molecular orbitals (OMOs)^27–29^

15 $$\varphi_{p}\left(R\right)=\underset{q}{\sum }S_{pq}^{-\frac{1}{2}}\left(R\right)\phi_{q}\left(R\right)$$

where

16 $$S_{pq}\left(R\right)=\left\langle \phi_{p}\left(R\right)\right|\phi_{q}\left(R\right)\rangle =\underset{\mu \nu }{\sum }C_{p\mu }C_{q\nu }\left\langle \chi_{p}\left(R\right)\right|\chi_{q}\left(R\right)\rangle$$

is the overlap between the unmodified molecular orbitals (UMOs) of eq 14 evaluated at **R**. By following the theory developed by Helgaker et al. in ref 28 we express the Hamiltonian in eq 11 in the OMO representation as

17 HOMO(R)=∑pqh̃pqOMO(R)Epq+12∑pqrsg̃pqrsOMO(R)epqrs+∑αωαbα†bα+∑α∑pqωα2(λα·(dOMO(R)−⟨dOMO(R)⟩))pq(bα+bα†)Epq+12∑α(λα·⟨dOMO(R)⟩)2

where the one- and two-electron dipole self-energy contributions are included in the terms ${\mathrm{h̃}}_{pq}^{\mathrm{OMO}}$ and ${\mathrm{g̃}}_{pqrs}^{\mathrm{OMO}}$ , respectively.

The integrals in eq 17 can be expressed in the unmodified molecular basis as

18 $${\mathrm{h̃}}_{pq}^{\mathrm{OMO}}\left(R\right)=\underset{mn}{\sum }S_{pm}^{-\frac{1}{2}}\left(R\right)S_{qn}^{-\frac{1}{2}}\left(R\right){\mathrm{h̃}}_{mn}\left(R\right)$$

19 $${\mathrm{g̃}}_{pqrs}^{\mathrm{OMO}}\left(R\right)=\underset{mntu}{\sum }S_{pm}^{-\frac{1}{2}}\left(R\right)S_{qn}^{-\frac{1}{2}}\left(R\right)S_{rt}^{-\frac{1}{2}}\left(R\right)S_{su}^{-\frac{1}{2}}\left(R\right){\mathrm{g̃}}_{mntu}\left(R\right)$$

20 $$d_{pq}^{\mathrm{OMO}}\left(R\right)=\underset{mn}{\sum }S_{pm}^{-\frac{1}{2}}\left(R\right)S_{qn}^{-\frac{1}{2}}\left(R\right)d_{mn}\left(R\right)$$

and similarly for the dipole integrals and their expectation values. The differentiation of eqs 18 and 19 with respect to the coordinate of atom *K* at the position **R**_*K*_ gives

21 $$\frac{d{\mathrm{h̃}}_{pq}^{\mathrm{OMO}}}{dR_{K}}=\frac{d{\mathrm{h̃}}_{pq}}{dR_{K}}-\frac{1}{2}\{\frac{dS}{dR_{K}},\mathrm{h̃}{\}}_{pq}$$

22 $$\frac{d{\mathrm{g̃}}_{pqrs}^{\mathrm{OMO}}}{dR_{K}}=\frac{d{\mathrm{g̃}}_{pqrs}}{dR_{K}}-\frac{1}{2}\{\frac{dS}{dR_{K}},\mathrm{g̃}{\}}_{pqrs}$$

where the one-index transformation terms^28^ are introduced

23 $$\{\frac{dS}{dR_{K}},\mathrm{h̃}{\}}_{pq}=\underset{m}{\sum }\left(\frac{dS_{pm}}{dR_{K}}{\mathrm{h̃}}_{mq}+\frac{dS_{qm}}{dR_{K}}{\mathrm{h̃}}_{mp}\right)$$

24 $$\{\frac{dS}{dR_{K}},\mathrm{g̃}{\}}_{pqrs}=\underset{m}{\sum }\left(\frac{dS_{pm}}{dR_{K}}{\mathrm{g̃}}_{mqrs}+\frac{dS_{qm}}{dR_{K}}{\mathrm{g̃}}_{pmrs}+\frac{dS_{rm}}{dR_{K}}{\mathrm{g̃}}_{pqms}+\frac{dS_{sm}}{dR_{K}}{\mathrm{g̃}}_{pqrm}\right)$$

This procedure is generalized to obtain higher derivatives of the Hamiltonian in eq 17 in terms of UMO-integral derivatives and one-index transformations integrals^28^ since they are required to obtain Hessian expression.

### 2.4 QED-HF Hessian Expression

To obtain the QED-HF Hessian expression, we first expand the QED-HF electronic energy as^27,28^

25 $$\mathrm{\varepsilon ̃}\left(R,\mathrm{\kappa ̃}\right)=Ẽ\left(R\right)+{\mathrm{\kappa ̃}}^{T}\mathrm{g̃}\left(R\right)+\frac{1}{2}{\mathrm{\kappa ̃}}^{T}\mathrm{F̃}\left(R\right)\mathrm{\kappa ̃}+O\left({\mathrm{\kappa ̃}}^{3}\right)$$

where we collected the optimization parameters in vector ***κ̃***. In eq 25, we introduced the QED-HF static energy *Ẽ*(**R**), electronic energy gradient ***g̃*** (**R**), and Hessian ***F̃*** (**R**). For a fixed geometry **R**, the optimization condition requires the energy in eq 25 to be stationary with respect to all orbital variations

26 $$\frac{\partial }{\partial \mathrm{\kappa ̃}}\mathrm{\varepsilon ̃}\left(R,\mathrm{\kappa ̃}\right)=\mathrm{g̃}\left(R\right)+\mathrm{F̃}\left(R\right)\mathrm{\kappa ̃}+O\left({\mathrm{\kappa ̃}}^{2}\right)=0$$

where ***κ̃*** = **0** is the solution for **R** = **R**_0_, with ***g̃*** (**R**_0_) = **0**. By using the condition in eq 26 we can eliminate the dependence of eq 25 on the ***κ̃*** parameters and obtain

27 $$\mathrm{\varepsilon ̃}\left(R\right)=Ẽ\left(R\right)-\frac{1}{2}{\mathrm{g̃}}^{T}\left(R\right){\mathrm{F̃}}^{-1}\left(R\right)\mathrm{g̃}\left(R\right)+O\left({\mathrm{g̃}}^{3}\right)$$

Here, the geometrical dependence is explicit and confined to the Hamiltonian integrals in eq 17. By differentiating eq 27 at the reference geometry, we obtain the expression of the QED-HF molecular gradient and Hessian

28 $$\frac{d\mathrm{\varepsilon ̃}}{dR_{K}}=\frac{dẼ}{dR_{K}}$$

29 $$\frac{d^{2}\mathrm{\varepsilon ̃}}{dR_{K}dR_{L}}=\frac{d^{2}Ẽ}{dR_{K}dR_{L}}-\frac{d{\mathrm{g̃}}^{T}}{dR_{K}}{\mathrm{F̃}}^{-1}\frac{d\mathrm{g̃}}{dR_{L}}$$

It is worth noting that the QED-HF molecular gradient in eq 28 only contains a static contribution, while the Hessian in eq 29 has a relaxation contribution due to the response of the electrons to changes in the nuclear coordinates, in accordance with the Born–Oppenheimer approximation. By following the theory outlined in refs 28,30,31 we can write the static contribution to the QED-HF Hessian in the UMO basis as

30 $$\begin{array}{ll} \frac{d^{2}E_{\mathrm{QED‐HF}}}{dR_{K}dR_{L}} & =\underset{pq}{\sum }\frac{d^{2}{\mathrm{F̃}}_{pq}}{dR_{K}dR_{L}}{\mathrm{\gamma ̃}}_{pq}-\frac{d^{2}S_{pq}}{dR_{K}dR_{L}}{\mathrm{F̃}}_{pq}\\ & -\frac{dS_{pq}}{dR_{K}}\frac{dY_{pq}}{dR_{L}}-\frac{dS_{pq}}{dR_{L}}\frac{dY_{pq}}{dR_{K}}+\frac{d^{2}V^{\mathrm{nuc}}}{dR_{K}dR_{L}}\\ & +\frac{1}{2}\underset{\alpha }{\sum }\frac{d^{2}\left(\lambda_{\alpha }\cdot \left\langle d\right\rangle \right)}{dR_{K}dR_{L}} \end{array}$$

where we introduced the auxiliary matrix

31 $$\frac{dY_{pq}}{dR_{K}}=\frac{d{\mathrm{F̃}}_{pq}}{dR_{K}}-\frac{1}{4}\{\frac{dS}{dR_{K}},\mathrm{F̃}{\}}_{pq}-\frac{1}{2}\underset{t}{\sum }\frac{dS_{pt}}{dR_{K}}{\mathrm{F̃}}_{tq}$$

with *F̃*_*pq*_ and *γ̃*_*pq*_ being the QED Fock matrix and the QED-HF density matrix, respectively. The relaxation contribution to the Hessian is written as

32 dg̃TdRKF̃−1dg̃dRL=∑ai⟨QED‐HF|[Eai−,(dHdRK)T]|QED‐HF⟩dκaidRL

where we used the notation

33 $$E_{ai}^{-}=E_{ai}-E_{ia}$$

to denote the singlet excitation operator. In eq 32, the orbital relaxation to geometric distortions is calculated by solving the linear system of equations

34 ∑bj⟨QED‐HF|[Eai,[Ebj−,H]]|QED‐HF⟩dκbjdRK=−⟨QED‐HF|[Eai,dHdRK]|QED‐HF⟩

for each nucleus and Cartesian component. The above response equations are solved iteratively using a linear subspace solver without explicitly constructing the electronic Hessian on the left-hand side. Note that both the static and relaxation parts of the Hessian in eqs 30 and 32 are similar to standard HF expressions, except for the presence of the dipole self-energy contributions, which are also included in the orbital relaxation of eq 34.

### 2.5 QED-HF Vibrational Dipole Strength

In the harmonic approximation, the dipole strength for the *n*-th fundamental vibrational transition is given by^32^

35 $$D_{n}=\frac{1}{2\omega_{n}}\left[P_{n}\cdot P_{n}\right]$$

where ω_*n*_ is the vibrational frequency of the *n*-th transition and **P**_*n*_ is the polar tensor in normal coordinates

36 $$P_{n}=\underset{K}{\sum }P_{K}L_{K,n}$$

where **L**_*K*,*n*_ is the Cartesian-to-normal coordinates transformation matrix and **P**_*K*_ are the components of the atomic polar tensor (APT) with respect to the coordinates of the *K*-th nucleus

37 $$P_{K}=E_{K}+N_{K}$$

where **E**_*K*_ and **N**_*K*_ denote the electronic and nuclear contributions, respectively. As discussed in ref 19 and building on the formalism in Section 2.4, the electronic contribution to the APT for polaritonic systems is expressed as

$$E_{K}=\underset{pq}{\sum }(\frac{dd_{pq}^{\mathrm{el}}}{dR_{K}}-\frac{1}{2}\{\frac{dS}{dR_{K}},d^{\mathrm{el}}{\}}_{pq})\;{\mathrm{\gamma ̃}}_{pq}-4\underset{ai}{\sum }\frac{d\kappa_{ai}}{dR_{K}}d_{ai}^{\mathrm{el}}$$ 38

where the orbital relaxation to geometric distortions is required. It is important to note that the QED contribution in eq 38 is included through both the density matrix and the orbital relaxation term. The pure nuclear contribution in eq 37 can be expressed as

39 $$N_{K}=Z_{K}1$$

where *Z*_*K*_ is the nuclear charge of the nucleus *K* and **1** is the identity matrix.

## 3 Validation and Implementation

The calculation of the QED-HF Hessian, vibrational frequencies, and dipole strengths has been implemented in a development version of the $e^{T}$ program.^33^ This implementation follows the standard procedure for the calculation of vibrational properties.^28^ The QED-HF Hessian code was validated by numerical differentiation of the QED-HF molecular gradient. The geometric derivatives of the integrals were calculated using phasedINT^34^ a library for computing Gaussian integrals in computational chemistry. The QED-HF vibrational dipole strength was validated by setting the coupling strength to zero and comparing the results with those obtained from the corresponding HF code.

## 4 Results and Discussion

The molecular geometries were optimized at the QED-HF level using the aug-cc-pVDZ basis set at the different coupling strengths. The code utilizes an analytical gradient and translational-rotational internal coordinates (TRIC)^35^ allowing for the inclusion of both internal coordinates and the rotational degrees of freedom required to account for the quantum electromagnetic field. Thus, we optimized both the molecular geometry and the orientation. This approach provides a lower bound for cavity-induced effects, since suboptimal orientations would lead to stronger interactions, as the dipole self-energy contribution, which is the only term that depends on the molecular orientation, is not minimized. The IR spectra shown below were obtained using Lorentzian distribution functions with a half-width at half-maximum (HWHM) of 10 cm^–1^. The intensities are normalized with respect to the intensity of the most intense peak overall. For all calculations, we employed coupling values of 0.00, 0.05, and 0.10 au. These relatively large couplings were chosen to better highlight the effects, even though such values are currently experimentally unfeasible.

### 4.1 Formaldehyde

The IR spectra of formaldehyde for coupling values of 0.00, 0.05, and 0.10 au are reported in Figure 1 for the fingerprint region and Figure 2 for the CH-stretching region, while the vibrational frequencies and the integrated intensities are listed in Table 2. The spectrum obtained in the absence of the quantum electromagnetic field is qualitatively in agreement with experimental data^36^ as shown for instance in ref 37.

**Figure 1:**
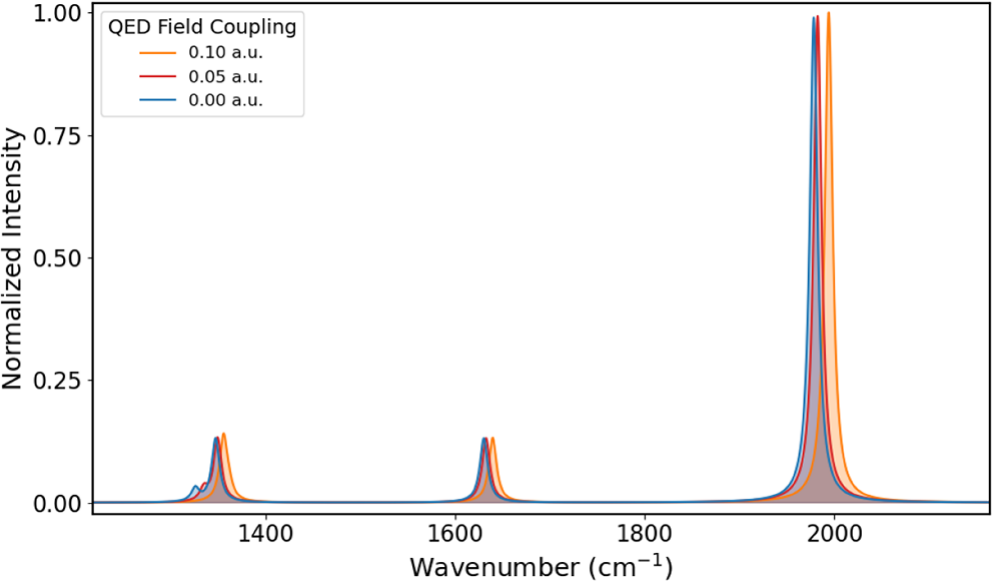
Fingerprint region of the IR spectrum of formaldehyde obtained for coupling values of 0.00, 0.05, and 0.10 au.

**Figure 2:**
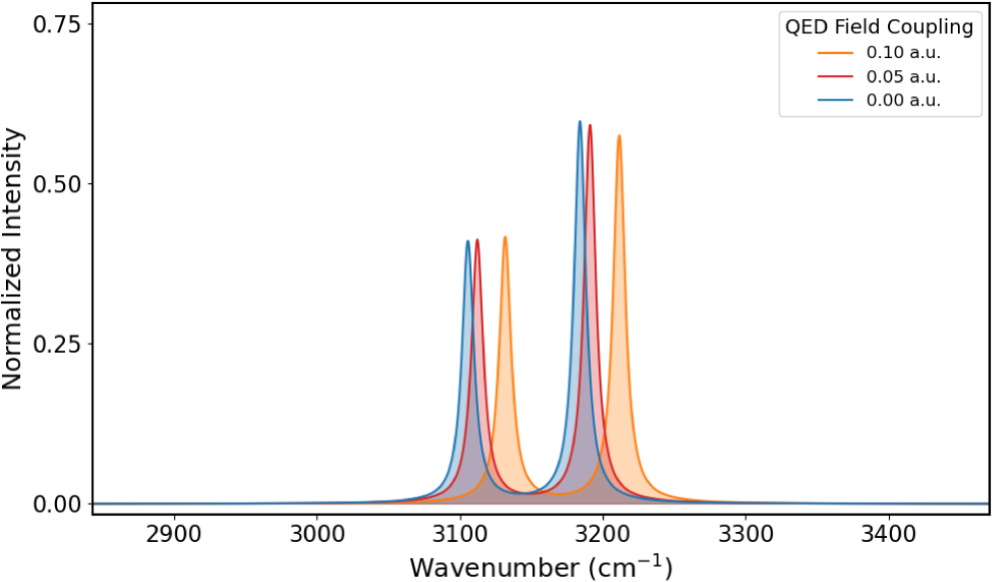
CH-stretching region of the formaldehyde IR spectrum obtained for coupling values of 0.00, 0.05, and 0.10 au.

As the coupling increases, we observe a blue shift for all bands, which is expected to be attributed to the contraction of the electronic density induced by the quantum electromagnetic field. Indeed, as shown in Figure 3, the electron density is compressed by the cavity, which results in a shortening of the bond distances (see Table 1).

**Figure 3:**
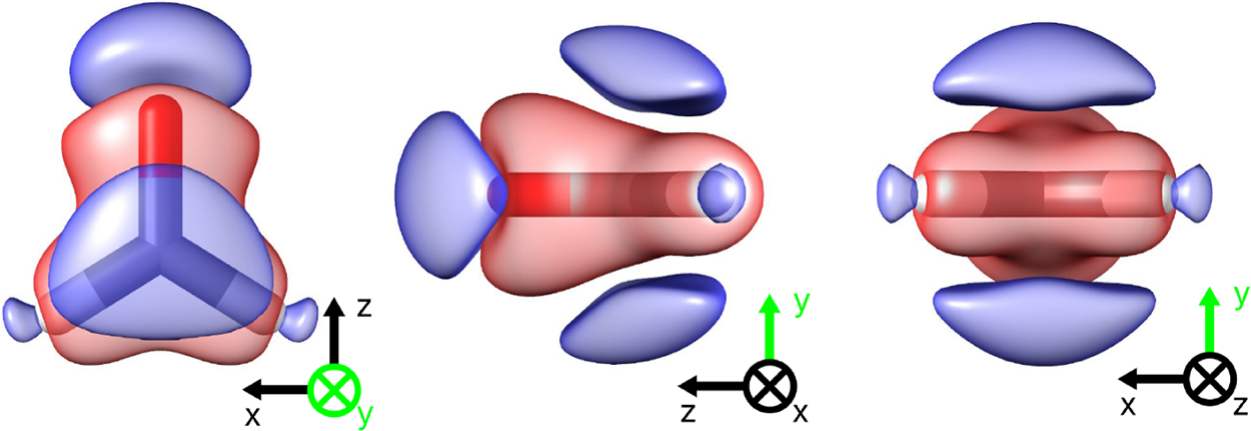
Electronic density difference in formaldehyde between couplings of 0.10 au and 0.00 au. Red regions denote electronic density increases, blue regions denote decreases. The isosurface value is set to 2 × 10^–4^ au.

**Table 1:** Bond-Length Differences Δ*r* (Å) Relative to the Quantum Field-Free Geometry at Different Coupling Strengths (au) in Formaldehyde

	0.05	0.10
Bond	Δ*r*	Δ*r*
C–O	–6.37 × 10^–4^	–2.44 × 10^–3^
C–H	–6.87 × 10^–4^	–2.72 × 10^–3^
C–H	–6.87 × 10^–4^	–2.72 × 10^–3^

Interestingly, as the coupling increases, the wagging vibrational mode becomes more destabilized compared to the rocking mode. At a coupling value of 0.10 au, the wagging mode becomes higher in energy than the rocking mode, as shown in Table 2. Because of the proximity of their energies, they appear as a single band centered at about 1355 cm^–1^, as shown in Figure 4.

**Figure 4:**
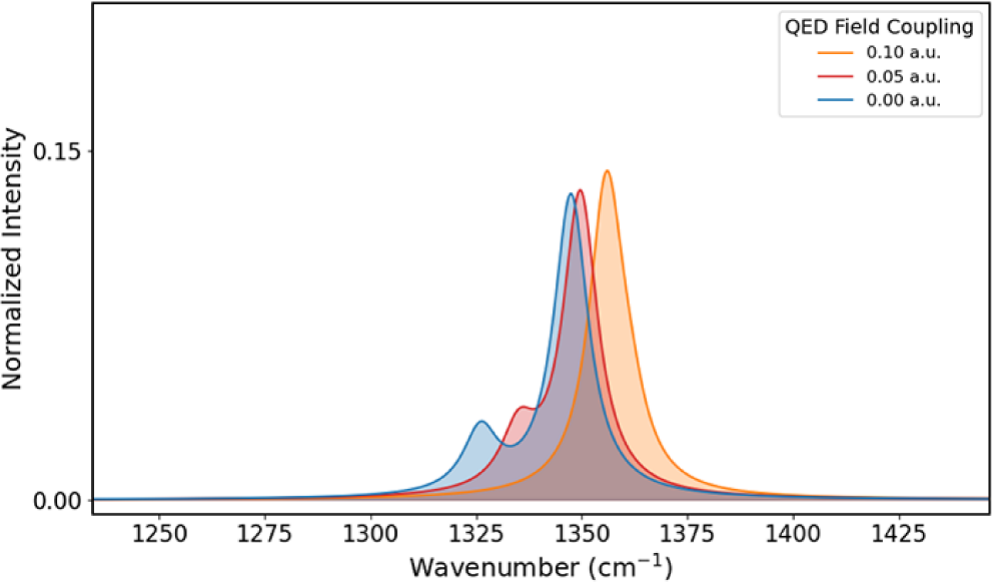
Region of the formaldehyde IR spectrum containing the wagging and rocking normal modes for coupling values of 0.00, 0.05, and 0.10 au.

**Table 2:** Vibrational Energies (cm^–1^) and IR Intensities (Km mol^–1^) of Formaldehyde at Different Coupling Strengths (au)

	0.00		0.05		0.10	
Assignment	*ν̃*	I	*ν̃*	I	*ν̃*	I
rotation	-	-	54.0	25.73	106.3	26.80
rotation	-	-	75.1	0.00	148.0	0.00
wagging	1326.1	4.24	1335.1	3.98	1361.2	3.31
rocking	1347.5	20.45	1349.7	20.50	1355.8	20.67
scissoring	1630.4	20.61	1632.8	20.64	1639.7	20.74
C=O stretching	1978.3	155.61	1982.5	156.07	1994.2	157.26
C–H stretching (sym.)	3105.7	64.21	3112.3	64.53	3131.8	65.29
C–H stretching (asym.)	3184.1	93.73	3191.2	92.82	3211.7	90.23

This behavior can be explained by the effect of the cavity on the electronic density. In the case of formaldehyde, the most stable configuration is the one in which the molecular dipole moment is orthogonal to the polarization of the electromagnetic field in the cavity. In this situation, the cavity confines the electronic density onto the molecular plane.^31^ As a result, vibrational modes that involve nuclear motions out of the molecular plane are inhibited, as in the case of wagging, depicted in Figure 5.

**Figure 5:**
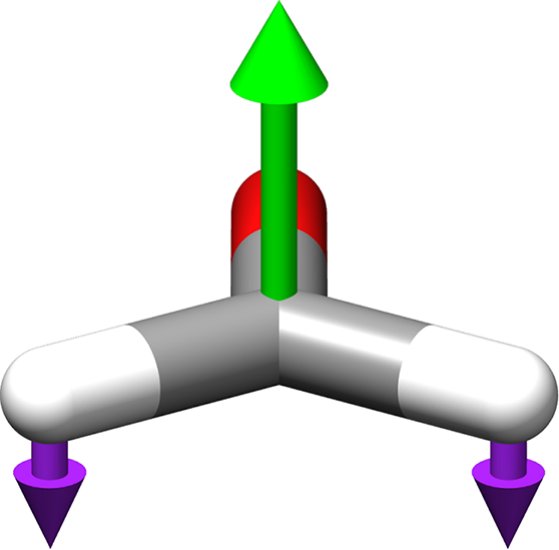
Wagging normal mode in formaldehyde. Purple arrows represent the nuclear displacements, and the green arrow represents the polarization orientation of the quantum electromagnetic field.

Another consequence is a reduction of the intensity as the nuclear displacements are restrained. Regarding the other normal modes, we observe an increase in intensity for the C=O stretching and the symmetric C–H stretching, and a decrease in intensity for the antisymmetric CH-stretching mode. Moreover, in Table 2 we observe two new low-frequency signals. The first corresponds to the rotation of formaldehyde around an axis orthogonal to both the polarization direction and the molecular dipole moment. The second also arises from a rotational mode. However, its intensity is null, as the associated rotation occurs around the molecular symmetry axis and does not induce a change in the dipole moment.

### 4.2 *p*-Nitroaniline

The IR spectrum of PNA at different coupling values of 0.00, 0.05, and 0.10 au is shown in Figure 6 from 450 to 1100 cm^–1^, Figure 7 from 1150 to 1900 cm^–1^, and Figure 8 from 3200 to 4000 cm^–1^. The corresponding vibrational energies and IR intensities are reported in Table 3. Comparison with experimental data in the absence of the cavity field shows qualitative agreement.^36^ In Figure 6, the band around 500 cm^–1^ corresponds to a wagging mode of the amino group, shown in Figure 9a. As the coupling increases, it becomes red-shifted while its intensity increases. This wagging mode corresponds to a nuclear displacement that drives the molecule toward a planar geometry. In the presence of a quantum electromagnetic field, the amino group becomes increasingly planar as the coupling strengthens.^38^ This suggests that displacements of the hydrogen atoms toward the molecular plane are energetically less penalized, indicating a possible flattening of the PES along these coordinates, and thus a lowering of the vibrational energy. In such a configuration, the derivatives of the dipole moment along the corresponding normal mode increase, leading to an enhancement in the intensity. In contrast, the out-of-plane wagging mode at 560 cm^–1^, shown in Figure 9b, is suppressed since it involves further distortion with the displacement of the hydrogen atoms away from the molecular plane, resulting in an increase of the frequency and a decrease in the intensity at higher couplings. Considering the modes related to the in- and out-of-plane deformations of the benzene moiety, whose vibrational energies are around 950 cm^–1^, they exhibit a behavior similar to what observed for formaldehyde. The lower-frequency mode in Figure 10a exhibits a larger shift than the other mode in Figure 10b. The former involves significant out-of-plane nuclear displacements, strongly restricted by the quantum electromagnetic field, whereas the latter, characterized by in-plane displacements, experiences a smaller frequency shift. Similar behavior can be observed in other regions of the IR spectrum at higher vibrational energies. This behavior could be attributed to bond strengthening induced by electron density contraction caused by the quantum electromagnetic field. Again, this result is in line with what was previously noted with formaldehyde.

**Table 3:** Vibrational Energies (cm^–1^) and IR Intensities (Km mol^–1^) of PNA in the Absence and Presence of the Quantum Electromagnetic Field at Different Couplings (au)

0.00		0.05		0.10	
*ν̃*	I	*ν̃*	I	*ν̃*	I
512.5	313.02	508.8	348.18	488.8	389.18
560.1	98.71	560.5	63.51	565.5	18.00
576.9	5.76	576.9	5.78	580.0	5.80
689.7	11.93	689.6	1.70	693.2	15.68
689.7	2.09	690.1	13.10	694.0	1.65
762.6	4.42	767.3	4.14	776.5	3.07
870.0	66.43	875.4	64.64	888.6	47.63
887.5	4.50	888.9	6.36	895.1	26.42
902.0	0.02	908.7	0.02	927.3	0.03
937.7	36.95	944.2	36.31	958.8	31.97
962.1	35.07	963.4	34.91	967.5	34.34
1070.0	1.19	1077.6	1.18	1091.0	1.65
1089.6	0.58	1090.5	0.64	1095.6	0.92
1093.7	0.01	1102.0	0.01	1119.2	0.01
1156.0	4.65	1155.8	4.26	1156.0	3.38
1203.9	6.27	1204.9	6.30	1210.6	6.50
1225.1	116.31	1226.5	118.81	1231.3	127.37
1288.7	17.50	1290.1	17.25	1296.0	16.03
1358.3	3.70	1357.4	3.68	1358.9	3.56
1399.4	116.73	1402.3	118.99	1411.3	125.60
1434.2	0.09	1436.0	0.08	1442.8	0.06
1580.7	2.05	1583.0	1.96	1591.5	2.03
1604.2	640.96	1606.0	647.58	1611.0	664.93
1657.0	9.52	1659.4	10.48	1668.4	13.93
1744.0	141.11	1746.4	140.61	1754.5	145.75
1770.4	61.67	1772.7	60.33	1781.2	48.53
1797.0	245.46	1799.1	248.74	1805.6	268.42
1814.2	426.00	1816.6	426.26	1824.5	420.41
3342.0	9.30	3346.9	9.90	3361.7	11.32
3342.7	11.73	3347.6	12.15	3362.6	13.16
3400.7	0.22	3406.0	0.37	3421.9	0.103
3400.7	3.13	3406.1	2.81	3421.9	2.73
3798.8	58.63	3807.0	59.68	3830.1	63.09
3910.3	30.85	3919.7	31.06	3946.3	31.89

**Figure 6:**
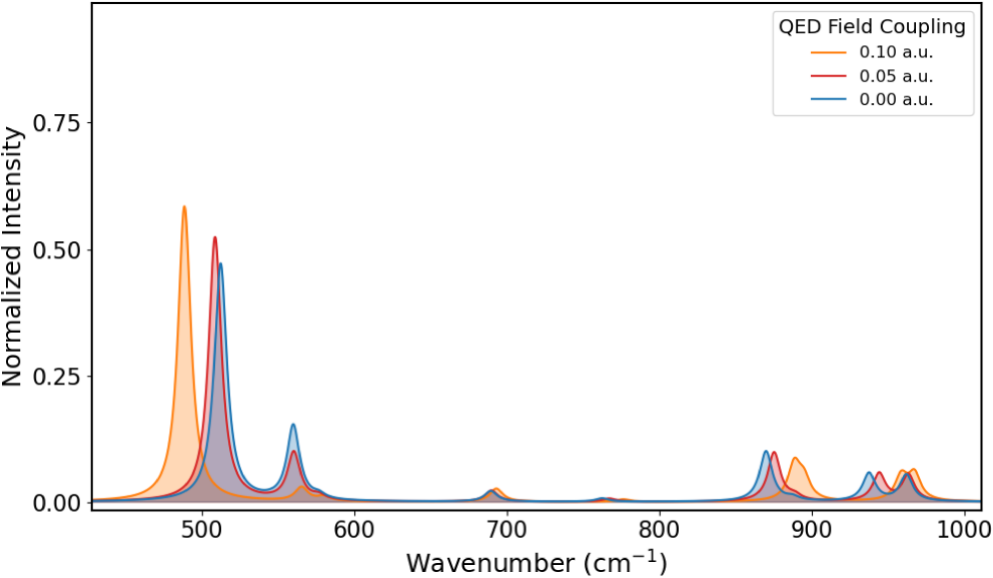
IR spectra of PNA from 450 to 1000 cm^–1^, obtained for coupling values of 0.00, 0.05, and 0.10 au.

**Figure 7:**
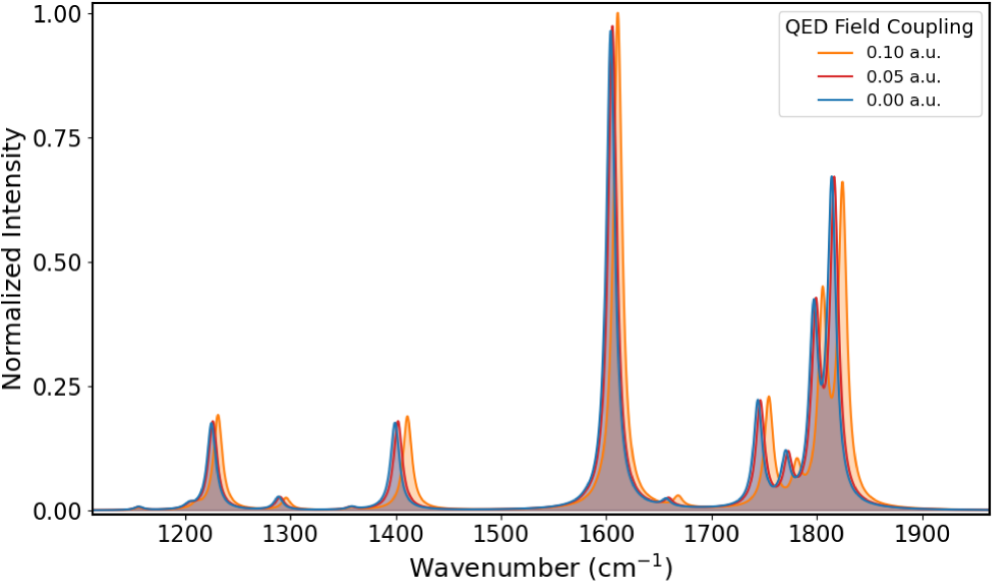
IR spectra of PNA in the 1150–1900 cm^–1^ region obtained for coupling values of 0.00, 0.05, and 0.10 au.

**Figure 8:**
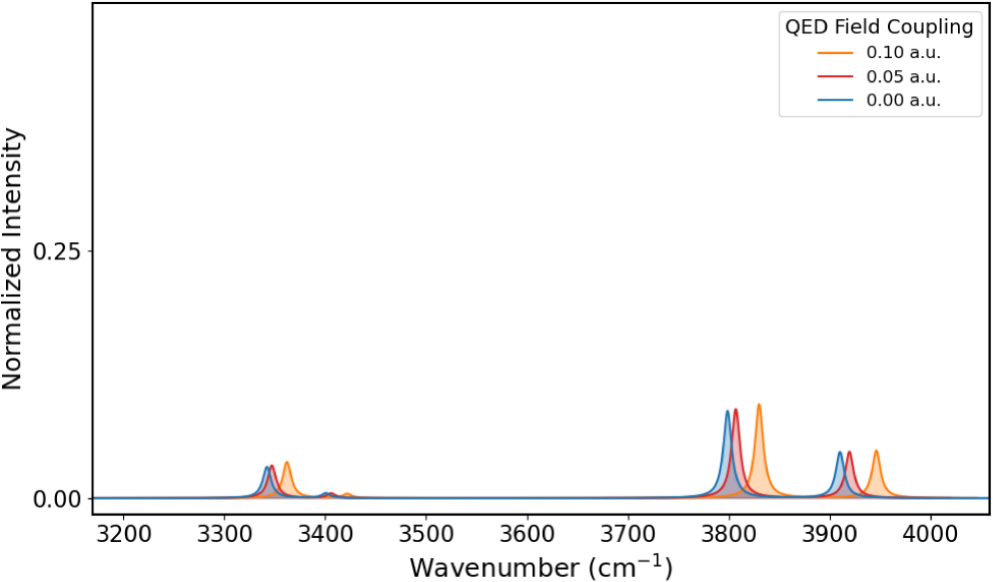
IR spectra of PNA in the 3200–4000 cm^–1^ region obtained for coupling values of 0.00, 0.05, and 0.10 au.

**Figure 9:**
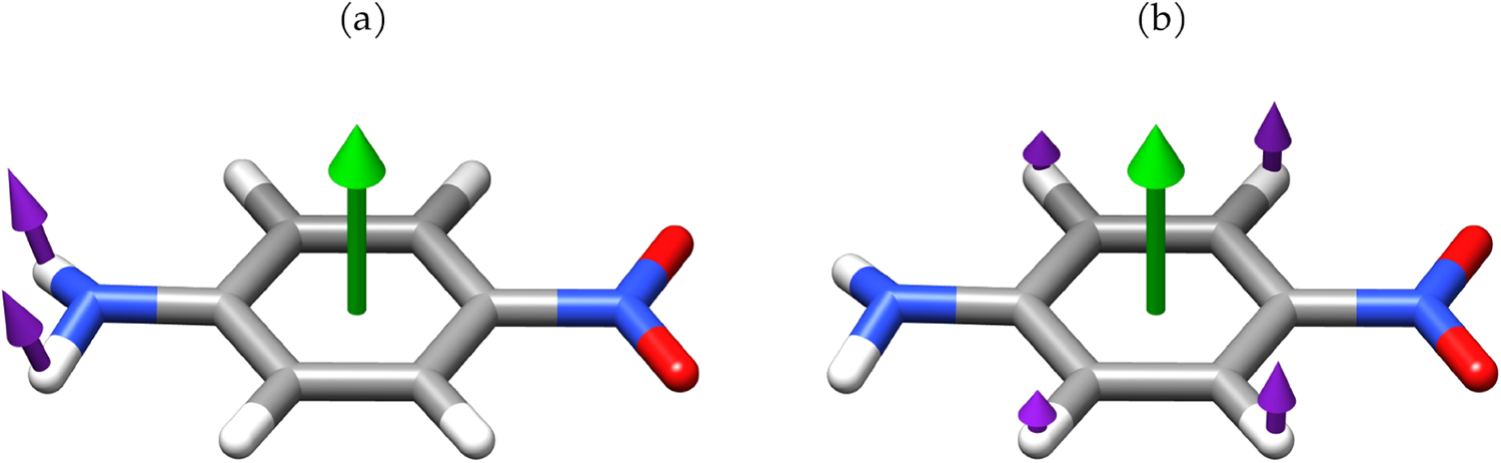
NH_2_ rocking (a) and out-of-plane molecular wagging mode (b) in PNA. Purple arrows show the nuclear displacements, while green arrows represent the polarization orientation of the quantum electromagnetic field.

**Figure 10:**
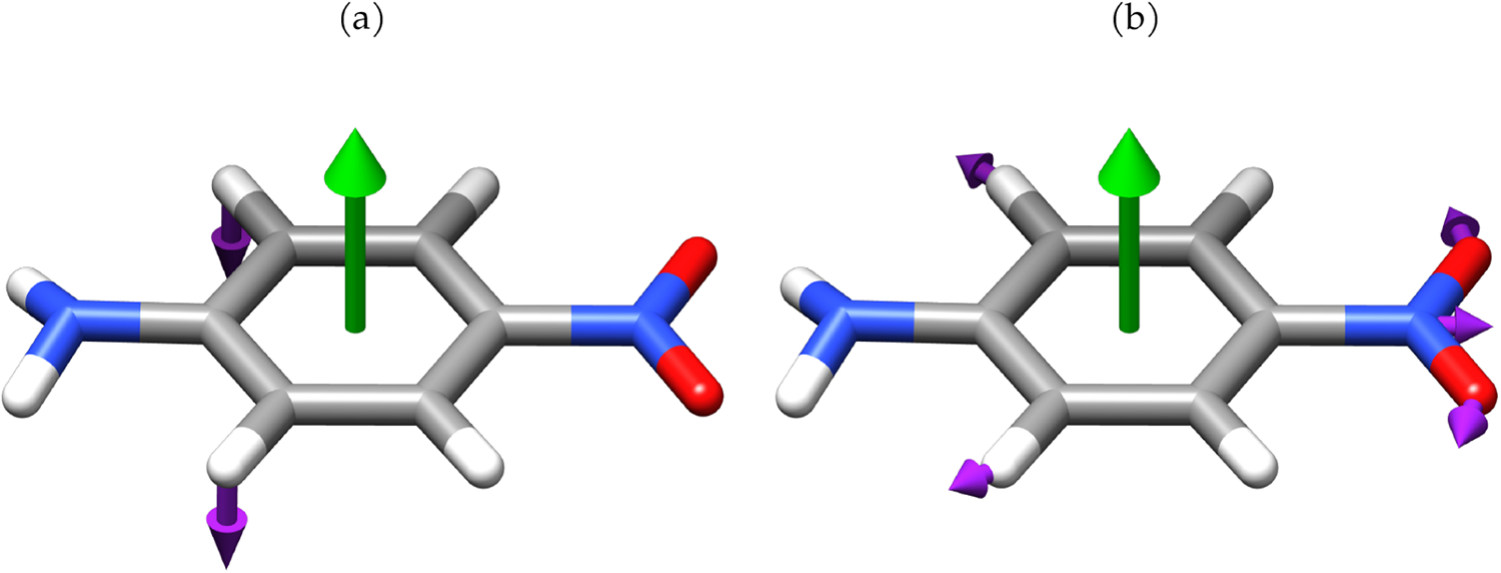
Normal modes associated with the transitions at 938 cm^–1^ (a) and 962 cm^–1^ (b) in PNA. Purple arrows show the nuclear displacements, while green arrows represent the polarization orientation of the quantum electromagnetic field.

### 4.3 Adamantane

As a final example, the IR spectra of adamantane are shown in Figure 11, while the predicted vibrational energies and IR intensities corresponding to allowed transitions are collected in Table 4. The results, in the absence of the quantum electromagnetic field, show qualitative agreement with previous investigations^39^ and experimental data.^36^

**Figure 11:**
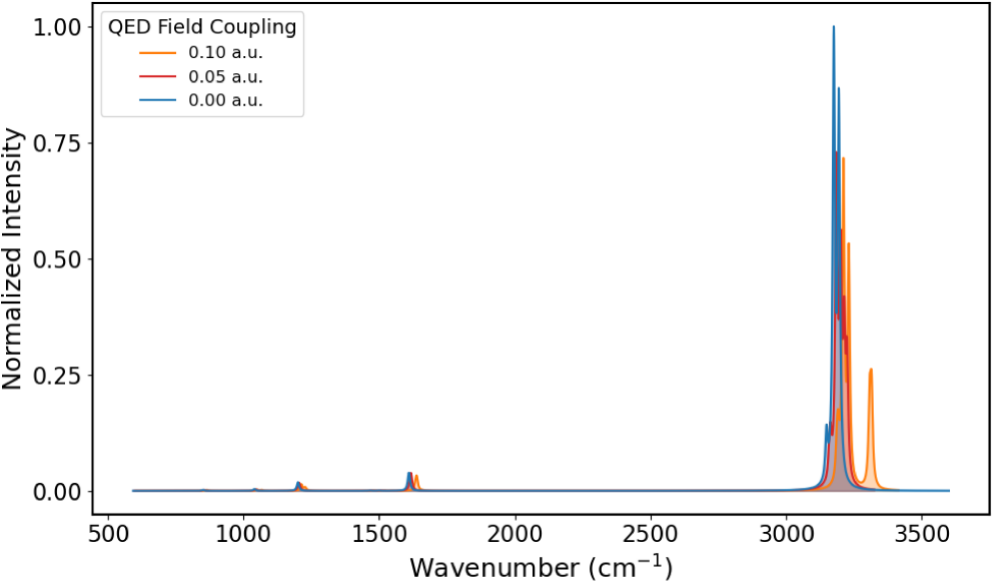
IR spectra of adamantane obtained for coupling values of 0.00, 0.05, and 0.10 au.

**Table 4:** Vibrational Energies (cm^–1^) and IR Intensities (Km mol^–1^) of Adamantane for Different Couplings (au)

0.00		
Irrep	*ν̃*	I
T_2_	850.0	0.39
T_2_	1200.1	3.51
T_2_	1609.5	7.50
T_2_	3175.7	181.05
T_2_	3148.8	19.37
T_2_	3194.7	154.20

0.05		
Irrep	*ν̃*	I
A_1_	853.1	0.41
E	854.0	0.39
E	1203.7	3.56
A_1_	1207.0	3.66
A_1_	1615.9	7.78
E	1617.0	7.33
E	3158.8	10.20
A_1_	3160.7	11.92
A_1_	3163.5	33.06
E	3165.7	3.46
E	3185.4	191.02
A_1_	3195.9	21.60
E	3201.1	124.17
E	3213.7	23.83
A_1_	3214.1	122.20
A_1_	3224.7	141.25

0.10		
Irrep	*ν̃*	I
A_1_	862.1	0.44
E	865.2	0.39
E	1213.4	3.79
A_1_	1226.5	3.92
A_1_	1634.4	8.42
E	1638.6	6.81
E	3185.6	6.70
A_1_	3188.8	36.93
A_1_	3192.6	33.17
E	3194.0	3.97
E	3210.9	195.14
A_1_	3222.8	0.0015
E	3231.0	140.76
E	3302.4	0.07
A_1_	3308.2	98.94
A_1_	3314.8	108.48

In the absence of the cavity field, the molecule has a *T*_d_ symmetry and only transitions to states of T_2_ symmetry are IR active. Once the cavity field is applied, the preferred orientation corresponds to the polarization aligned along one of the C_3_ axes. This causes a slight contraction along that axis and a lowering of the symmetry to the C_3*v*_ group. The allowed transitions are now those leading to a final state of A_1_ or E symmetry. Due to the cavity field-driven symmetry breaking, the T_1_ and T_2_ transitions are split into A_2_ and E, and A_1_ and E transitions, respectively. The vibrational energies with non-null IR intensities are reported in Table 4 for coupling values of 0.05 and 0.10 au, and the IR spectrum in the CH-stretching region is shown in Figure 12.

**Figure 12:**
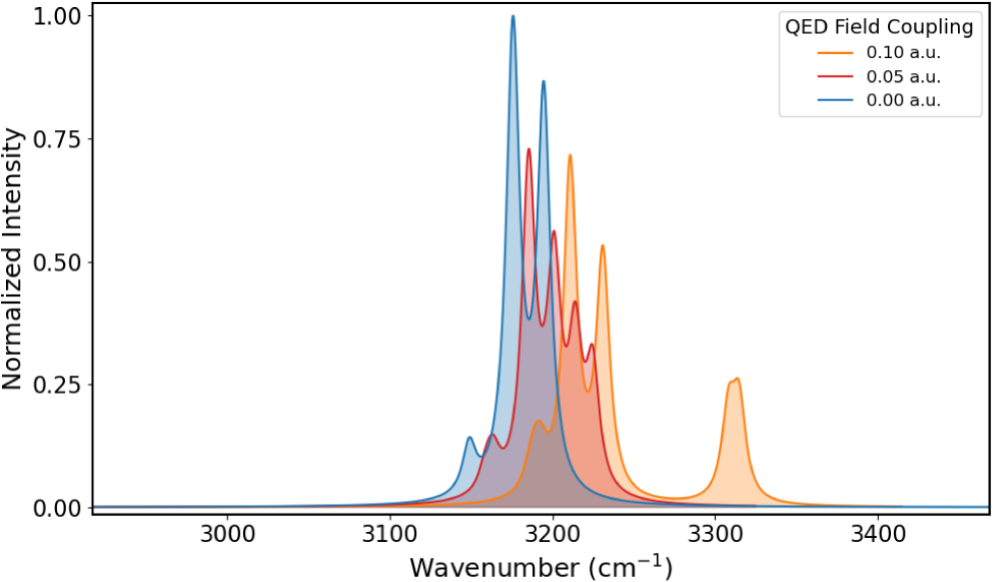
IR spectra in the CH-stretching region of adamantane obtained for coupling values of 0.00, 0.05, and 0.10 au.

As the coupling with the quantum electromagnetic field increases, nuclear displacements along the polarization axis become more restrained. This is expected to cause a decrease in the intensity, as observed for the A_1_ transition at 3315 cm^–1^. This happens when the change in the dipole moment is generated by a nuclear displacement aligned with the quantum electromagnetic field, as shown in Figure 13a for the aforementioned transition. In contrast, when nuclear displacements are along other directions, the intensities could be enhanced by the cavity, as observed for the transition to the state of E symmetry at 3211 cm^–1^, whose normal modes are shown in Figure 13b.

**Figure 13:**
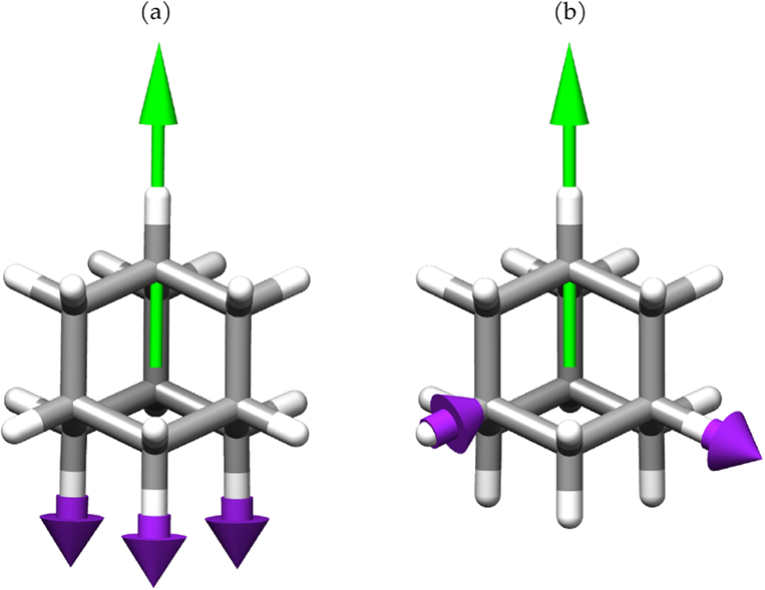
Normal modes corresponding to the transitions at 3211 cm^–1^ (a) and 3231 cm^–1^ (b) for adamantane at a coupling of 0.10 au. Purple arrows indicate nuclear displacements, while green arrows represent the polarization orientation of the quantum electromagnetic field.

Figure 14a presents the Duschinsky matrix for the C–H region, calculated in the absence of the cavity field and with a coupling strength of 0.10 au. The matrix shows a significant redistribution of the normal modes induced by the cavity. To provide a physical understanding of this mode mixing, we consider the simplest case of the A_1_ symmetry modes, which have frequencies of 3222.8, 3308.2, and 3314.8 cm^–1^, shown in Figure 15d–f, respectively. These modes overlap with T_2_ symmetry modes of frequencies of 3175.7, 3192.3, and 3194.7 cm^–1^ obtained in the absence of the cavity, reported in Figure 15a–c. As can be observed, the effect of the cavity leads to a rearrangement of such modes, resulting in a separation of nuclear motions parallel and orthogonal to the cavity field. The influence of the coupling strength on mode mixing is illustrated in Figure 14b. The near-diagonal form of the corresponding Duschinsky matrix demonstrates an almost one-to-one correspondence between the normal modes obtained at 0.05 and 0.10 au, indicating that further increases in the coupling do not produce additional modifications.

**Figure 14:**
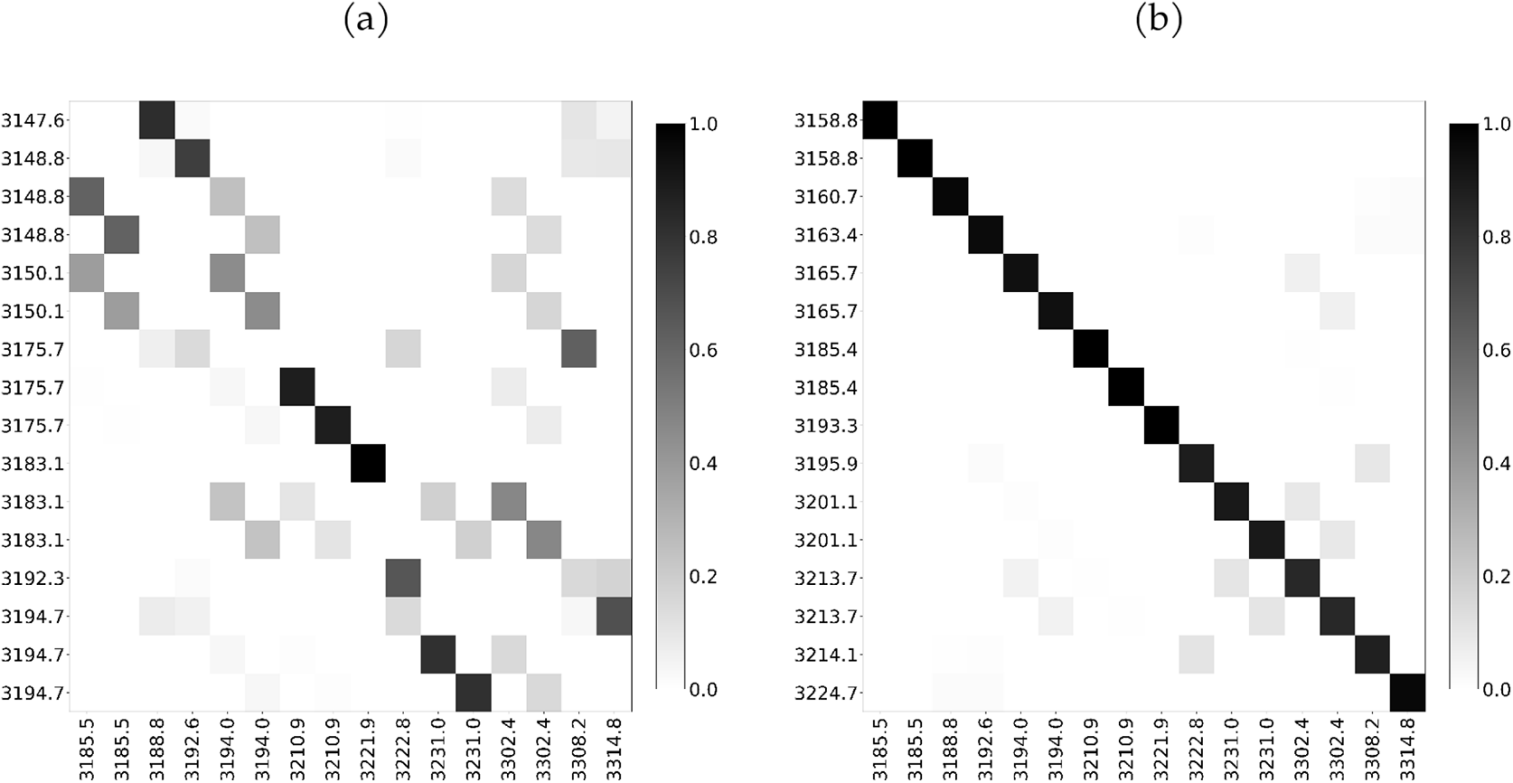
Duschinsky matrices for the normal modes in the CH-stretching region of adamantane. (a) Comparison between the molecule outside the cavity and the molecule coupled to a cavity with a coupling strength of 0.10 au. (b) Comparison between the molecule coupled to a cavity at 0.05 au and 0.10 au coupling strength. Each mode is labeled by its vibrational frequency (cm^–1^). The normal modes corresponding to the configuration with the lower coupling strength are always shown on the *y*-axis of the matrices. To display each matrix, its elements were first squared, and a shade of gray was associated from white (0) to black (1).

**Figure 15:**
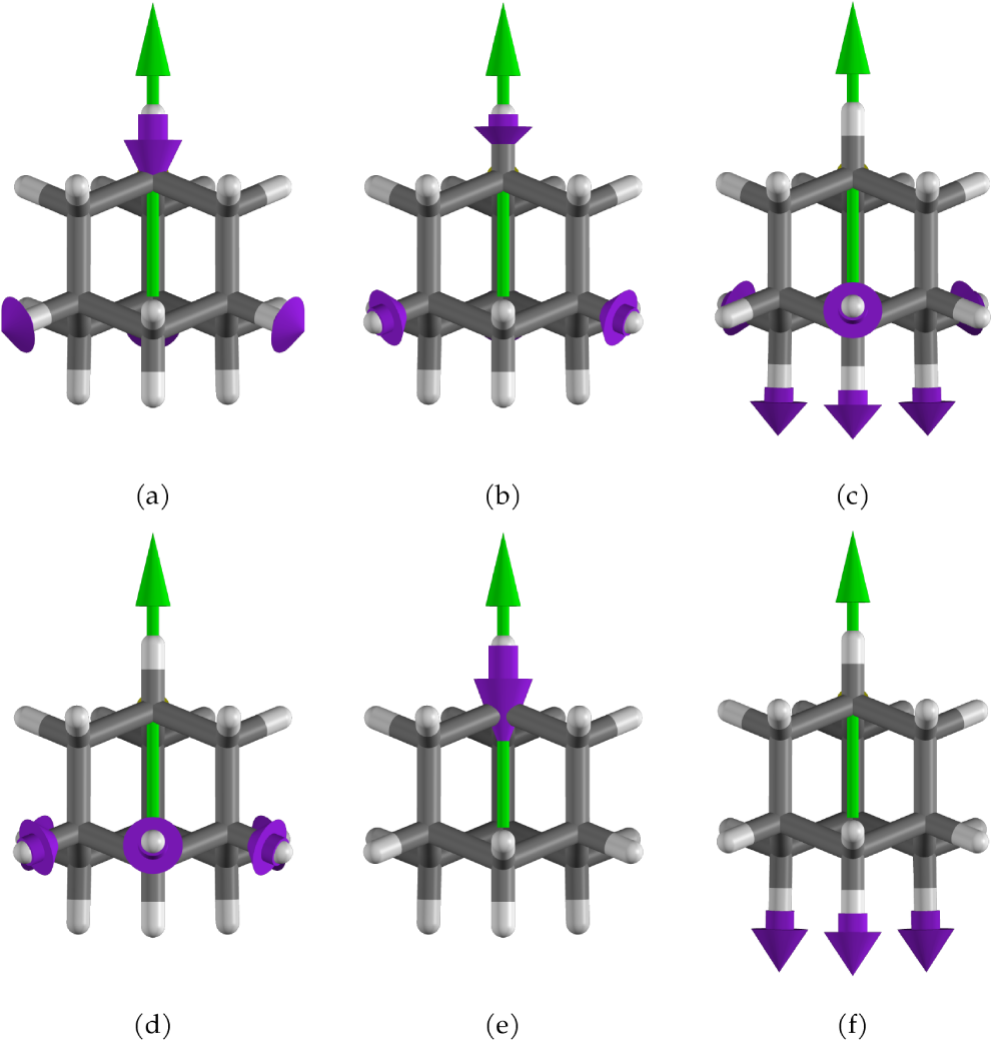
Panels (a–c) show normal modes computed without the cavity field. Panels (d–f) show normal modes computed with a cavity field of coupling strength 0.10 au, corresponding to A_1_ symmetry transitions.

Finally, in Figure 12, we note that there is a frequency increase for all transitions due to the contraction of the electronic density induced by the quantum electromagnetic field, as previously observed with formaldehyde and PNA.

We also investigated the influence of two orthogonal cavity modes, each with a coupling of 0.05 au, to more faithfully reproduce the experimental setup.^40^ Figure 16a,b presents the comparison with the single-mode spectrum at a coupling of 0.05 au, and Table 5 summarizes the corresponding vibrational frequencies and intensities.

**Figure 16:**
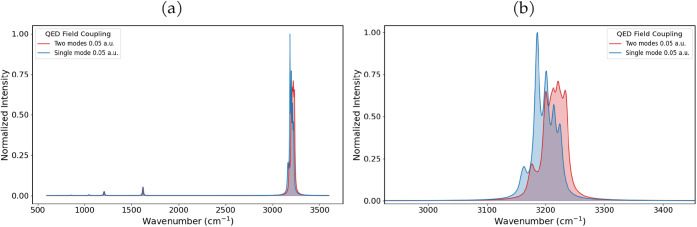
(a) IR spectra and (b) IR spectra in the CH-stretching region of adamantane obtained for a single mode with coupling 0.05 au and two orthogonal modes, each with coupling 0.05 au.

**Table 5:** Vibrational Energies (cm^–1^) and IR Intensities (Km mol^–1^) of Adamantane with Two Modes, Each with Coupling 0.05 au

Two-mode cavity 0.05			
*ν̃*	I	*ν̃*	I
857.4	0.41	3174.3	5.92
857.7	0.41	3176.8	46.10
858.5	0.39	3182.8	14.50
1045.9	0.74	3187.1	8.38
1048.9	0.83	3199.9	206.20
1050.7	0.85	3208.2	99.69
1207.8	3.58	3213.0	69.35
1210.0	3.70	3213.6	57.55
1211.0	3.67	3217.7	19.43
1623.0	7.68	3220.9	92.20
1623.9	7.30	3221.5	61.77
1624.8	7.48	3226.5	85.94
3172.5	8.83	3231.9	58.52
3173.8	12.51	3234.6	161.12

Introducing two orthogonal modes further reduces the molecular symmetry, leading to a complete lifting of the degeneracy of the vibrational modes. All bands show a blue shift, which is consistent with the cavity-induced electron density contraction and bond strengthening. The two-mode coupling leads to an enhanced mixing among the C–H normal modes.

This effect is highlighted by the Duschinsky matrix (Figure 17), where the off-diagonal elements increase in magnitude, indicating a stronger mode transformation.

**Figure 17:**
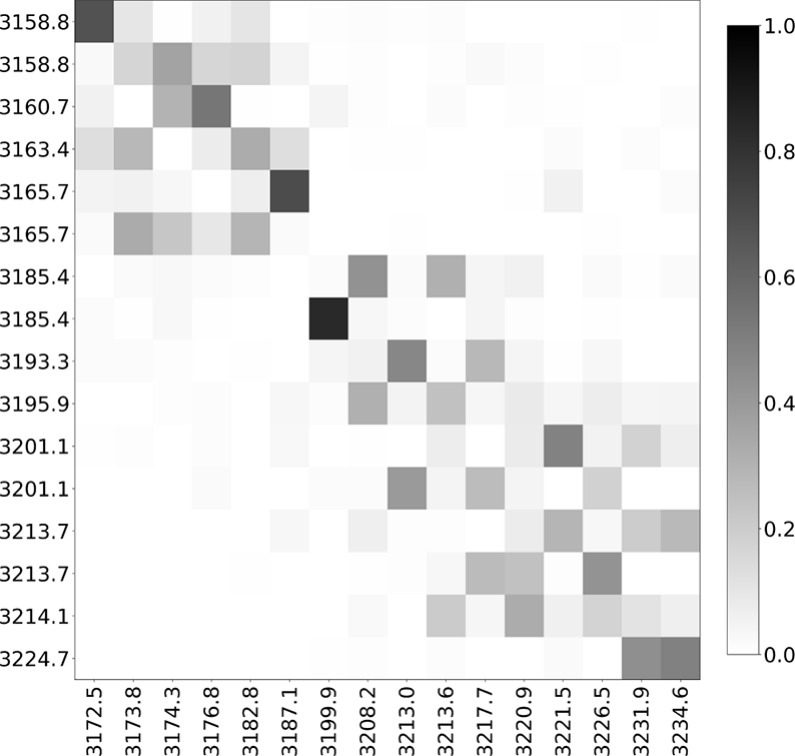
Duschinsky matrix for the CH-stretching normal modes of adamantane between a single cavity mode with coupling 0.05 au (vertical axis) and two orthogonal cavity modes, each with coupling 0.05 au (horizontal axis).

## 5 Conclusions

In this study, we presented the derivation and implementation of the analytical QED-HF Hessian and explored the influence of the quantum electromagnetic field on molecular vibrational properties. We observed significant modifications in vibrational frequencies, intensities, and normal modes induced by ESC. These changes can be attributed to the contraction of electron density, driven by the quantum electromagnetic field. In the case of planar systems, the normal modes involving out-of-plane nuclear displacements showed blue-shifted frequencies and reduced intensities. As observed in formaldehyde, the combination of such effects causes the wagging and rocking vibrational modes to merge into a single band. In PNA, the out-of-plane modes follow the same behavior. In contrast, the wagging vibrational mode of the amino group shows a reduction in frequency and a substantial increase in intensity. Strong coupling also impacts molecular symmetry, as observed in adamantane. The symmetry reduction from T_*d*_ to C_3*v*_ induced by the quantum electromagnetic field enables previously forbidden IR transitions to become allowed and leads to the splitting of bands. Moreover, the analysis of the Duschinsky matrices reveals that coupling to the cavity field induces significant changes in the normal modes in the C–H stretching region, leading to a separation of nuclear motions between those parallel and orthogonal to the cavity polarization. However, this effect does not increase substantially with higher coupling strengths above 0.05 au, implying that further increases have a negligible effect on the vibrational modes. The inclusion of two orthogonal cavity modes at 0.05 au further reduces the molecular symmetry, fully lifting the vibrational degeneracies, and further transforming the C–H normal modes. Overall, these findings demonstrate that strong coupling significantly influences vibrational frequencies, intensities, and molecular symmetry.

Furthermore, the analytical evaluation of the Hessian is more efficient and stable than numerical differentiation, with a computational cost equivalent to that of a standard HF Hessian calculation. This enables the calculation of higher-order derivatives, which are required, for instance, for the study of anharmonic effects that will be explored in future work. Finally, the response formalism developed to evaluate the QED-HF Hessian, could be extended to time-dependent QED-approaches^30^ enabling the investigation of spectroscopic properties of polaritonic excited states in the ESC regime.

## Data Availability

The code used to obtain the findings of this study is available from the corresponding author upon reasonable request. The supporting data are publicly available in ref 38.

## References

[1] Pulay P. (1969). Ab initio
calculation of force constants and equilibrium geometries in polyatomic
molecules. Mol. Phys..

[2] Bishop D. M., Randic M. (1966). Ab Initio Calculation
of Harmonic Force Constants. J. Chem. Phys..

[3] Stanton J. F., Gauss J. (2000). Analytic second derivatives
in high-order many-body perturbation
and coupled-cluster theories: Computational considerations and applications. Int. Rev. Phys. Chem..

[4] Handy N. C., Amos R. D., Gaw J. F., Rice J. E., Simandiras E. D. (1985). The elimination
of singularities in derivative calculations. Chem. Phys. Lett..

[5] Harrison R. J., Fitzgerald G. B., Laidig W. D., Barteltt R. J. (1986). Analytic
MBPT­(2)
second derivatives. Chem. Phys. Lett..

[6] Jørgensen P., Helgaker T. (1988). Møller-Plesset
energy derivatives. J. Chem. Phys..

[7] Helgaker T., Jørgensen P., Handy N. C. (1989). A numerically stable procedure for
calculating Møller-Plesset energy derivatives, derived using
the theory of Lagrangians. Theor. Chim. Acta.

[8] Koch H., Jensen H. J. A., Jørgensen P., Helgaker T., Scuseria G. E., Schaefer H. F. (1990). Coupled cluster
energy derivatives. Analytic Hessian
for the closed-shell coupled cluster singles and doubles wave function:
Theory and applications. J. Chem. Phys..

[9] Kállay M., Gauss J. (2004). Analytic second derivatives for general coupled-cluster and configuration-interaction
models. J. Chem. Phys..

[10] Gauss J., Stanton J. F. (1997). Analytic CCSD­(T)
second derivatives. Chem. Phys. Lett..

[11] Gauss J., Stanton J. F. (2000). Analytic first and
second derivatives for the CCSDT-n
(n = 1–3) models: a first step towards the efficient calculation
of CCSDT properties. Phys. Chem. Chem. Phys..

[12] Bonini J., Flick J. (2022). Ab Initio Linear-Response
Approach to Vibro-Polaritons in the Cavity
Born-Oppenheimer Approximation. J. Chem. Theory
Comput..

[13] Fischer E. W., Syska J. A., Saalfrank P. (2024). A Quantum Chemistry Approach to Linear
Vibro-Polaritonic Infrared Spectra with Perturbative Electron-Photon
Correlation. J. Phys. Chem. Lett..

[14] Fiechter M. R., Richardson J. O. (2024). Understanding
the cavity Born–Oppenheimer approximation. J. Chem. Phys..

[15] Angelico S., Haugland T. S., Ronca E., Koch H. (2023). Coupled cluster cavity
Born–Oppenheimer approximation for electronic strong coupling. J. Chem. Phys..

[16] Haugland T. S., Ronca E., Kjønstad E. F., Rubio A., Koch H. (2020). Coupled Cluster
Theory for Molecular Polaritons: Changing Ground and Excited States. Phys. Rev. X.

[17] Ruggenthaler M., Sidler D., Rubio A. (2023). Understanding polaritonic chemistry
from ab initio quantum electrodynamics. Chem.
Rev..

[18] Fregoni J., Corni S., Persico M., Granucci G. (2020). Photochemistry in the
strong coupling regime: A trajectory surface hopping scheme. J. Comput. Chem..

[19] Schnappinger T., Kowalewski M. (2023). Ab Initio Vibro-Polaritonic Spectra in Strongly Coupled
Cavity-Molecule Systems. J. Chem. Theory Comput..

[20] Schnappinger T., Sidler D., Ruggenthaler M., Rubio A., Kowalewski M. (2023). Cavity Born-Oppenheimer
Hartree-Fock Ansatz: Light-Matter Properties of Strongly Coupled Molecular
Ensembles. J. Phys. Chem. Lett..

[21] Huang X., Liang W. (2025). Analytical derivative
approaches for vibro-polaritonic structures
and properties. I. Formalism and implementation. J. Chem. Phys..

[22] Lexander M. T., Angelico S., Kjønstad E. F., Koch H. (2024). Analytical Evaluation
of Ground State Gradients in Quantum Electrodynamics Coupled Cluster
Theory. J. Chem. Theory Comput..

[23] Lexander, M. T. ; M. Trabski, J. H. ; Bianchi, A. ; Kjønstad, E. F. ; Haugland, T. S. ; Koch, H. Exploring Equilibrium Geometries in Static and Quantized Fields. In Preparation. 2025. 10.1021/acs.jctc.5c00739 PMC12573757 41055955

[24] Mandal A., Montillo Vega S., Huo P. (2020). Polarized Fock states and the dynamical
Casimir effect in molecular cavity quantum electrodynamics. J. Phys. Chem. Lett..

[25] Frisk
Kockum A., Miranowicz A., De Liberato S., Savasta S., Nori F. (2019). Ultrastrong coupling between light
and matter. Nat. Rev. Phys..

[26] Rokaj V., Welakuh D. M., Ruggenthaler M., Rubio A. (2018). Light–matter
interaction in the long-wavelength limit: no ground-state without
dipole self-energy. J. Phys. B: Mol. Opt. Phys..

[27] Helgaker T. U., Almlöf J. (1984). A second-quantization
approach to the analytical evaluation
of response properties for perturbation-dependent basis sets. Int. J. Quantum Chem..

[28] Helgaker T. U., Almlöf J., Jensen H. J. A., Jørgensen P. (1986). Molecular
Hessians for large-scale MCSCF wave functions. J. Chem. Phys..

[29] Olsen J., Bak K. L., Ruud K., Helgaker T., Jörgensen P. (1995). Orbital connections
for perturbation-dependent basis sets. Theoret.
Chim. Acta.

[30] Castagnola M., Riso R. R., Barlini A., Ronca E., Koch H. (2024). Polaritonic
response theory for exact and approximate wave functions. Wiley Interdiscip. Rev.: Comput. Mol. Sci..

[31] Barlini A., Bianchi A., Ronca E., Koch H. (2024). Theory of Magnetic
Properties in Quantum Electrodynamics Environments: Application to
Molecular Aromaticity. J. Chem. Theory Comput..

[32] Bak K. L., Jørgensen P., Helgaker T., Ruud K., Jørgen
Aa. Jensen H. (1994). Basis set convergence of atomic axial tensors obtained
from self-consistent field calculations using London atomic orbitals. J. Chem. Phys..

[33] Folkestad S. D., Kjønstad E. F., Myhre R. H., Andersen J. H., Balbi A., Coriani S., Giovannini T., Goletto L., Haugland T. S., Hutcheson A. (2020). e T 1.0: An open source electronic structure
program with emphasis on coupled cluster and multilevel methods. J. Chem. Phys..

[34] Bianchi, A. ; Ronca, E. ; Koch, H. PhasedInt: efficient integral evaluation for gaussian basis functions with complex phase support. In Preparation. 2025.

[35] Wang L.-P., Song C. (2016). Geometry optimization made simple with translation and rotation coordinates. J. Chem. Phys..

[36] NIST Mass Spec Data Center, d., S.E. Stein In NIST Chemistry WebBook, NIST Standard Reference Database Number 69, Linstrom, P. J. ; Mallard, G. ; National Institute of Standards and Technology:Gaithersburg MD, 2015, p 20899.

[37] Leszczynski J., Goodman L., Kwiatkowski J. (1997). Density functional
theory and post-Hartree-Fock
studies on molecular structure and harmonic vibrational spectrum of
formaldehyde. Theor. Chem. Acc..

[38] Barlini, A. ; Bianchi, A. ; M. Trabski, J. H. ; Bloino, J. ; Koch, H. Supporting data for Cavity Field-Driven Symmetry Breaking and Modulation of Vibrational Properties: Insights from the Analytical QED-HF Hessian. 2025; 10.5281/zenodo.15291382. PMC12529899 41042097

[39] Bistričić L., Pejov L., Baranović G. (2002). A density functional theory analysis
of Raman and IR spectra of 2-adamantanone. J.
Mol. Struct.:THEOCHEM.

[40] Jayachandran A., Patrahau B., Ricca J. G., Mahato M. K., Pang Y., Nagarajan K., Thomas A., Genet C., Ebbesen T. W. (2025). A Phenomenological
Symmetry Rule for Chemical Reactivity Under Vibrational Strong Coupling. Angew. Chem., Int. Ed..

